# Diagnostic Challenge of Pediatric Gaucher Disease in a Low‐Resource South Asian Setting: A Case Report

**DOI:** 10.1002/ccr3.73346

**Published:** 2026-08-17

**Authors:** Muhammad Waqas, Rahila Bano, Muhammad Usman Haider, Kumar Abhishek, Arshad Mehmood, Aqeel Abbas, Abbas Ali, Talha Basheer, Rafiya Muhammad Altaf, Yahya Samadi

**Affiliations:** ^1^ Gomal Medical College Dera Ismail Khan Pakistan; ^2^ Geisinger Health System Wilkes‐Barre Pennsylvania USA; ^3^ HCA Centerpoint Medical Center Kansas City Missouri USA; ^4^ Jinnah Hospital Lahore Pakistan; ^5^ Karachi Institute of Medical Sciences Karachi Pakistan; ^6^ Dow International Medical College Karachi Pakistan; ^7^ Kateb University Kabul Afghanistan

**Keywords:** consanguinity, Gaucher disease, glucosylceramidase/deficiency, hepatosplenomegaly, pancytopenia

## Abstract

Gaucher disease is a rare autosomal recessive lysosomal storage disorder that is caused by a deficiency of the enzyme “β‐glucocerebrosidase”, leading to the accumulation of glucocerebroside within macrophages. It commonly presents with hepatosplenomegaly, cytopenias, and bone marrow infiltration. Gaucher disease is a pan‐ethnic lysosomal storage disorder reported worldwide. Although few cases have been described in Pakistan and other South Asian countries, limited awareness and restricted access to diagnostic testing may contribute to underdiagnosis and delayed recognition in this region. We report the case of a 2‐year‐old girl who presented with fever and cough of 1 week's duration. Her mother reported normal birth and development until 6 months of age, after which she developed progressive weakness and abdominal distension. She had a history of recurrent hospitalizations for anemia, chest infections, and abdominal swelling. Family history revealed consanguinity and early childhood deaths of two siblings and one cousin with similar complaints. The clinical examination revealed pallor, weakness, and marked hepatosplenomegaly. A complete blood count showed features of pancytopenia; therefore, a bone marrow examination was advised, which demonstrated characteristic Gaucher cells, supporting a presumptive diagnosis of Gaucher disease. Symptomatic treatment was initiated, and the patient was referred to Children Hospital, Lahore, Pakistan.

## Key Clinical Message

Gaucher disease may present in infants with unexplained hepatosplenomegaly and cytopenias. In low‐resource settings, reliance solely on bone marrow morphology, without confirmatory enzymatic or genetic testing, can delay diagnosis, subtyping, and the timely initiation of appropriate therapy.

**Abbreviations**
CNS: Central nervous system
ERT: Enzyme replacement therapy
GBA1: Glucosylceramidase beta 1 gene
HCT: Hematocrit
LD bodies: Leishman‐Donovan bodies
MCH: Mean corpuscular hemoglobin
MCHC: Mean corpuscular hemoglobin concentration
MCV: Mean corpuscular volume
ME ratio: Myeloid‐to‐erythroid ratio
MP: Malaria parasite
RBC: Red blood cell count
RDW: Red cell distribution width
SRT: Substrate reduction therapy
WBC: White blood cell count

## 1 Introduction

Gaucher disease is an autosomal recessive lysosomal storage disorder caused by mutations in the glucosylceramidase beta 1 (GBA1) gene, located on chromosome 1 (1q21). These mutations lead to the deficiency of the enzyme β‐glucocerebrosidase (glucosylceramidase or acid β‐glucosidase), which converts glucocerebroside into ceramide and glucose. Failure of conversion results in the accumulation of glucocerebroside within macrophages and multiple organs [1].

Although Gaucher disease is well documented in developed countries, its incidence and prevalence are significantly lower in Asia and Pakistan. Several studies from Pakistan and neighboring South Asian countries have reported Gaucher disease with diverse genetic mutations, suggesting that the condition is underdiagnosed rather than truly rare in this region. Limited awareness, absence of newborn screening programs, and restricted access to molecular diagnostics contribute to delayed recognition. Reported incidence in Asia‐Pacific is around 1.24–1.36 per 100,000 live births [2]; no reliable national incidence and prevalence data are available from Pakistan.

The majority of patients present in childhood with progressive abdominal distension, recurrent infections, fatigue, and growth retardation. Their clinical manifestations include hepatosplenomegaly, pancytopenia (anemia, thrombocytopenia, leukopenia), and skeletal involvement such as bone pain, crises, or fractures [3]. Central nervous system involvement is absent in Type 1 Gaucher disease, while its involvement is characteristic of Type 2 and Type 3 [4].

Therefore, we report a presumptive case of Gaucher disease in a 2‐year‐old Pakistani girl with progressive clinical deterioration, recurrent infections, hepatosplenomegaly, and a notable family history of consanguinity and early childhood deaths. This case highlights the diagnostic challenges encountered in low‐resource South Asian settings, as well as the importance of bone marrow examination where access to advanced diagnostic tools remains limited. Our case also highlights the importance of studying Gaucher disease in different ethnic groups.

### 1.1 Case History/Examination

This is a case report of a 2‐year‐old girl who presented to the Pediatric Department of Mufti Mehmood Teaching Hospital, Dera Ismail Khan, Pakistan, with chief complaints of fever and dry cough for 1 week. The fever was intermittent and high‐grade, associated with chills, and partially relieved with over‐the‐counter paracetamol, with no identifiable aggravating factors. The cough was non‐productive, persistent throughout the day, and not associated with wheezing, dyspnea, or chest pain.

According to the mother, the child had normal birth and development until 6 months of age, after which she developed progressive weakness and abdominal distension. She had been hospitalized 3–4 times previously for anemia, recurrent chest infections, progressive weakness, and abdominal swelling.

On general examination, the child was vitally stable but appeared pale and weak, with no dysmorphic features. Abdominal examination revealed marked hepatosplenomegaly, with the liver palpable approximately 4 cm below the right costal margin in the mid‐clavicular line and the spleen palpable 12–14 cm below the left costal margin extending toward the iliac fossa. There was no ascites or palpable abdominal mass.

Respiratory examination showed a symmetrical chest with normal movements and vesicular breath sounds bilaterally, without wheezing, stridor, crepitations, or signs of respiratory distress. Cardiovascular examination was unremarkable. Neurological examination revealed normal tone, reflexes, and age‐appropriate motor responses, with no abnormal eye movements, decreased blink rate, feeding difficulty, or bulbar signs. No lymphadenopathy was noted.

### 1.2 Differential Diagnosis

Hematological malignancy was considered because of the pancytopenia and hepatosplenomegaly; however, the peripheral smear showed no immature or blast cells, and bone marrow examination did not demonstrate malignant cells. Visceral leishmaniasis and malaria were considered because of the fever, cytopenias, and hepatosplenomegaly; however, no malaria parasites or Leishman‐Donovan bodies were identified. Hemoglobinopathy was also considered given the anemia and family history, but hemoglobin electrophoresis was unremarkable. The presence of numerous histiocytes with morphology consistent with Gaucher cells, together with the clinical features, supported a presumptive diagnosis of Gaucher disease.

### 1.3 Investigations

**FIGURE 1:**
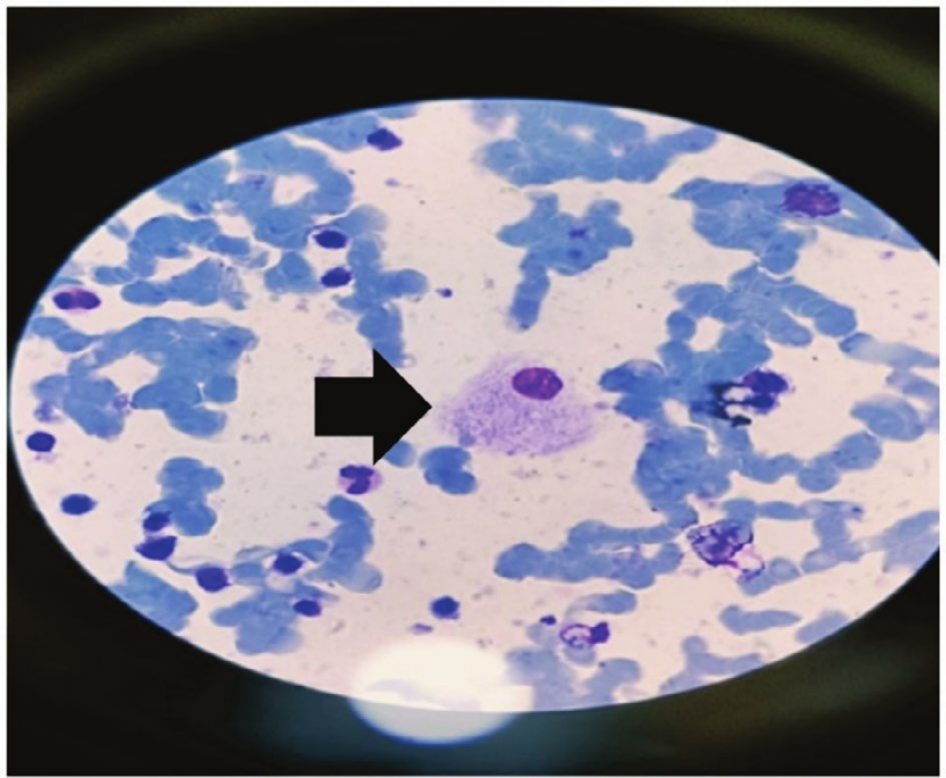
Bone marrow biopsy (bone marrow biopsy showing Gaucher cells).

Complete blood count and peripheral blood smear revealed pancytopenia (Table 1). Bone marrow aspiration and biopsy were subsequently performed, revealing increased histiocytes with morphology consistent with Gaucher cells (Table 2, Figure 1). Additional investigations were performed to exclude alternative diagnoses and assess organ involvement. Liver and renal function tests and Widal test were within normal limits. Hemoglobin electrophoresis and electrocardiography were unremarkable. Abdominal ultrasonography demonstrated hepatosplenomegaly without other significant abnormalities.

**TABLE 1:** Complete blood count and peripheral smear.

Parameter	Patient's value	Pediatric reference range (2 years)
Hemoglobin	6.8 g/dL	11.0–13.5 g/dL
WBC	3900/mm^3^	6000–17,000/mm^3^
Platelets	95,000/mm^3^	150,000–450,000/mm^3^
RBC	2.6 million/mm^3^	3.9–5.3 million/mm^3^
HCT	28.1%	33%–39%
MCV	82 fL	70–86 fL
MCH	22 pg.	23–31 pg.
MCHC	32 g/dL	30–36 g/dL
RDW	13.8%	11.5%–14.5%
Neutrophils	34%	30%–55%
Lymphocytes	58%	45%–70%
Monocytes	5%	2%–10%
Eosinophils	3%	0%–6%
Reticulocyte count	0.6%	0.5%–2%
Abnormal RBC cells	Anisopoikilocytosis (+), Microcytosis (+), Hypochromia (+), Tear drop cells (+)	—
Malaria parasite	No MP seen	—
**Remarks**	**Pancytopenia; no immature cells seen**	—

*Note:* Complete blood count and Peripheral smear.

**TABLE 2:** Bone marrow cytology.

Parameter	Findings
Site	Right tibia
Cellularity	Fairly cellular marrow with fragments and trails
Erythropoiesis	Active, megaloblastic
Myelopoiesis	Active, showing normal maturation
M:E Ratio	1:2
Megakaryopoiesis	Normal
Platelets	Decreased
Plasma cells	Not increased
Lymphocytes	Not increased
Abnormal cells	Increased histiocytes showing morphology of Gaucher cells
Hemoparasites	No MP/LD bodies
Iron stain	—
Special stain	—
**Remarks**	**Findings suggestive of Gaucher disease**

*Note:* Bone marrow cytology revealed increased histiocytes showing morphology of Gaucher cells.

Due to limited therapeutic resources in our setting, the patient was managed symptomatically. The patient was referred to a tertiary care center for confirmatory enzymatic testing and further management; however, no follow‐up information regarding biochemical confirmation, genetic testing, or initiation of enzyme replacement therapy was available at the time of manuscript preparation.

## 2 Discussion

Gaucher disease is a hereditary lysosomal storage disorder caused by a deficiency of the enzyme β‐glucocerebrosidase [2]. It is a pan‐ethnic disorder that has been reported worldwide across diverse populations. Several studies from India and other South Asian countries have documented Gaucher disease with considerable clinical and genetic heterogeneity, suggesting that the condition may be underdiagnosed rather than truly rare in this region [5, 6].

Gaucher disease is classified into three clinical subtypes: Type 1 (non‐neuronopathic), Type 2 (acute neuronopathic), and Type 3 (chronic neuronopathic). Type 2 typically presents in infancy with rapid neurological deterioration, including feeding difficulty, bulbar dysfunction, stridor, abnormal eye movements, decreased blink rate, and spasticity. Type 3 presents later in childhood and may demonstrate isolated slowing of horizontal saccadic eye movements. In our patient, although progressive weakness and early onset were noted, no detailed neurological abnormalities were documented. However, absence of formal neurological assessment limits definitive subtyping. In the absence of confirmatory enzymatic or genetic testing and adequate long‐term neurological follow‐up, the specific clinical subtype cannot be determined. Therefore, no definitive subtype classification was assigned in this case.

GD typically presents with anemia, thrombocytopenia, hepatosplenomegaly, and bone involvement. These features were also present in our patient and are consistent with findings from international records [4]. However, in many Asian reports, disease is not diagnosed until significant organomegaly or skeletal complications have developed because of limited awareness and diagnostic challenges [3, 7].

Gaucher disease is typically diagnosed by demonstrating deficient β‐glucocerebrosidase activity, with molecular analysis of the GBA1 gene providing supportive genetic confirmation. Bone marrow examination is neither required nor recommended as a primary diagnostic tool for Gaucher disease when enzyme assay or genetic testing is available. Diagnosis should ideally be confirmed by demonstrating reduced β‐glucocerebrosidase activity, which can even be performed using a dried blood spot test. In our setting, however, limited access to biochemical and molecular testing necessitated reliance on bone marrow morphology [3].

Enzyme replacement therapy (ERT) is the gold‐standard treatment due to proven benefits in improving blood counts, reducing organ size, and preventing skeletal complications [3, 8]. Substrate reduction therapy (SRT) may be considered in selected patients; however, it is generally not recommended as first‐line therapy in pediatric patients. Enzyme replacement therapy remains the standard of care for children with Gaucher disease. Unfortunately, in countries like Pakistan, where ERT and SRT are not usually available, management is mainly supportive. This usually includes blood transfusions for anemia, antibiotics for infections, and splenectomy [3, 9]. Splenectomy may improve cytopenia but carries long‐term risks and does not prevent disease progression in bones and other organs.

## 3 Conclusion

Our case report highlights the importance of considering Gaucher disease in the differential diagnosis of children presenting with unexplained hepatosplenomegaly and cytopenias, even in regions where the condition is rarely reported, such as Pakistan and South Asia. The lack of access to advanced diagnostic tools and disease‐specific therapies in low‐resource countries significantly affects patient outcomes. Highlighting this case not only emphasizes the diagnostic challenges in such contexts but also contributes to expanding awareness of Gaucher disease across diverse ethnic populations and emphasizes the need for improved diagnostic capacity in low‐resource settings.

## 4 Limitations

A significant limitation in this case is the absence of confirmatory enzyme assay or GBA1 mutation analysis. Gaucher cells on bone marrow examination are not pathognomonic and may be seen in pseudo‐Gaucher states such as chronic myeloid leukemia or thalassemia. Therefore, without biochemical or genetic confirmation, the diagnosis remains presumptive. This reflects the diagnostic constraints in low‐resource settings.

## Author Contributions

**Rahila Bano:** conceptualization, investigation, writing – original draft. **Kumar Abhishek:** writing – original draft, writing – review and editing, project administration, supervision. **Aqeel Abbas:** writing – original draft, writing – review and editing, data curation. **Muhammad Waqas:** conceptualization, investigation, formal analysis, data curation, supervision, writing – original draft, writing – review and editing. **Muhammad Usman Haider:** writing – original draft, supervision, data curation, writing – review and editing. **Rafiya Muhammad Altaf:** data curation, investigation, writing – original draft, methodology. **Arshad Mehmood:** writing – review and editing, formal analysis, data curation. **Abbas Ali:** data curation, investigation, writing – review and editing. **Yahya Samadi:** validation, visualization, software, resources, funding acquisition. **Talha Basheer:** writing – original draft, data curation.

## Funding

The authors have nothing to report.

## Ethics Statement

The authors have nothing to report.

## Consent

Written informed consent for publication was obtained from the patient's parent/legal guardian.

## Conflicts of Interest

The authors declare no conflicts of interest.

## Data Availability

The data that support the findings of this study are available on request from the corresponding author. The data are not publicly available due to privacy or ethical restrictions.
